# Pharmacological aspects of galantamine for the treatment of Alzheimer's disease

**DOI:** 10.17179/excli2016-820

**Published:** 2017-01-10

**Authors:** Jae Kwang Kim, Sang Un Park

**Affiliations:** 1 Division of Life Sciences, College of Life Sciences and Bioengineering, Incheon National University, Incheon, 406-772, Korea; 2 Department of Crop Science, Chungnam National University, 99 Daehak-ro, Yuseong-gu, Daejeon, 34134, Korea

## ⁯

Dear Editor,

Galantamine is a natural product belonging to the isoquinoline alkaloid family of compounds. It was first discovered and isolated in the 1950s from *Galanthus nivalis* (common snowdrop) and *Galanthus woronowii* (Caucasian snowdrop), members of the Amaryllidaceae family (Marco and do Carmo Carreiras, 2006[20]).

Alzheimer's disease (AD) is named after Dr. Alois Alzheimer, who first identified the disease in 1906. AD slowly destroys memory and thinking skills and is the most frequently diagnosed age-related neurodegenerative disorder (Prvulovic et al., 2010[25]). Galantamine is an acetylcholinesterase (AChE) inhibitor and one of the most promising drugs available for the treatment of AD and various other memory impairments (Scott and Goa, 2000[28]; Ago et al., 2011[1]). Synthetic galantamine was first approved for the treatment of AD in Sweden in 2000 and was subsequently approved in the European Union and the United States (Heinrich and Lee Teoh, 2004[13]). In the present report, we reviewed the most recent studies on the pharmacological activity of galantamine (Table 1(Tab. 1)) (References in Table 1: Hager et al., 2016[10]; Hishikawa et al., 2016[14]; Hwang et al., 2016[15]; Wahba et al., 2016[30]; Hanafy et al., 2016[12]; Inden et al., 2016[16]; Bezerra et al., 2016[4]; Misra et al., 2016[21];Oka et al., 2016[23]; Tokuchi et al., 2016[29]; Wu et al., 2015[32]; Nakano et al., 2015[22]; Atanasova et al., 2015[3]; Fornaguera et al., 2015[8]; Woo et al., 2015[31]; Bhattacharya et al., 2015[6]; Jiang et al., 2015[17]; Prins et al., 2014[24]; Alexandrova et al., 2014[2]; Furukawa et al., 2014[9]; Caramelli et al., 2014[7]; Koola et al., 2014[19]; Kita et al., 2014[18]; Hager et al., 2014[11]; Bhattacharya et al., 2014[5]; Richarz et al., 2014[27]; Ramakrishnan et al., 2014[26]).

| Key findings | Reference |
|---|---|
| No beneficial effects of galantamine therapy were observed in patients co-treated with memantine 2 years post treatment. The reasons for memantine treatment and the possibility of interactions between memantine and galantamine merit further investigation. | Hager et al., 2016 |
| Combination therapies using galantamine and cilostazol as well as the respective monotherapies maintained or even improved cognitive functions, affective functions, and activities of daily living functions in AD patients with asymptomatic lacunar infarction. | Hishikawa et al., 2016 |
| Switching cholinesterase inhibitor (ChEI) drugs is clinically feasible for non-responding patients with mild-to-moderate AD. Inclusion of galantamine in a switched group was as efficacious at maintaining cognition as that observed in a naïve group. | Hwang et al., 2016 |

| Key findings | Reference |
|---|---|
| The use of rod-like hydroxyapatite particles for selective delivery of galantamine drug and nanoceria may become an extremely powerful method for drug delivery to affected brain areas of patients with AD. | Wahba et al., 2016 |
| Nasal galantamine hydrobromide/chitosan complex nanoparticles have been shown to be pharmacologically efficacious. Further, their safety has been demonstrated *in vivo*, confirming their potential to contribute to the intranasal management of AD. | Hanafy et al., 2016 |
| Galantamine enhances striatal dopamine release through allosteric modulation of α4 nicotinic acetylcholine receptors on nigrostriatal dopaminergic terminals. | Inden et al., 2016 |
| Donepezil, tacrine, galantamine, and rivastigmine are known to cause convulsions and have anticholinesterase effects. Further, they have been shown to decrease locomotion in an invertebrate model. | Bezerra et al., 2016 |
| The bioavailability of galantamine hydrobromide-loaded solid-lipid nanoparticles is twice that of galantamine hydrobromide alone. Thus, these nanoparticle carriers show promise for safe and effective drug delivery, especially in diseases such as AD. | Misra et al., 2016 |
| Galantamine therapy, unlike donepezil, is characterized by a dual mechanism of action that may increase acetylcholine and the nicotinic receptor-modulation effect within the frontal lobe, both of which are associated with apathy and executive dysfunction in AD patients. | Oka et al., 2016 |
| Benefits to cognitive and affective functions were greater in AD patients receiving the combination therapy of galantamine plus ambulatory cognitive rehabilitation than in those receiving galantamine therapy only. | Tokuchi et al., 2016 |
| Galantamine may be involved in modifying AD pathophysiological mechanisms by alleviating amyloid-β deposition and neuroinflammation. The results from this study provide new evidence for the use of galantamine in the treatment of AD. | Wu et al., 2015 |
| This is a long-term study examining the efficacy of galantamine in very elderly AD patients, suggesting improved efficacy in male patients and baseline lower cognitive, affective, and activity of daily living functions. | Nakano et al., 2015 |
| A galantamine derivative is emerging as a promising lead compound for multi-target anti-AD therapy because of its strong inhibitory activity and ability to block amyloid beta deposition on acetylcholinesterase. | Atanasova et al., 2015 |
| Novel galantamine-loaded polymeric nanoparticles have been designed for the first time using a nano-emulsification approach. This study demonstrates the appropriate features required for advanced drug delivery systems to treat neurodegenerative diseases. | Fornaguera et al., 2015 |
| The patch system (galantamine hydrobromide loaded gel drug reservoirs in transdermal patches) has moderate pH, high drug content, and a controlled drug-release pattern. Thus, the patch system has the potential to be used as a drug carrier system for the treatment of AD. | Woo et al., 2015 |
| Memogain, a pro-drug, releases galantamine in the brain by cleavage with a carboxyesterase. Nasal applications of memogain effectively deliver the drug to the brain of AD patients with the potential to retard plaque deposition and improve behavioral symptoms like those observed with galantamine treatment. | Bhattacharya et al., 2015 |
| Galantamine significantly improves cognitive, behavioral, and global performance in patients with AD. However, it needs to be used with caution in clinical settings. | Jiang et al., 2015 |
| Patients with mild cognitive impairment treated with galantamine had a lower rate of whole brain atrophy, but not hippocampal atrophy, over a 24-month treatment period than patients who were treated with placebo. However, the protective effect of galantamine on the rate of whole brain atrophy in MCI was only observed in apolipoprotein E ϵ4 carriers. | Prins et al., 2014 |

| Key findings | Reference |
|---|---|
| Soman is a nerve agent that reduces hippocampal glutamatergic synaptic transmission and may result in cognitive deficits long after an acute exposure. Prevention of soman-induced reductions in hippocampal glutamatergic synaptic transmission may be an important determinant of galantamine's ability to counter the related cognitive deficits. | Alexandrova et al., 2014 |
| Pre-treatment with galantamine in a newborn rat model of hypoxia-ischemia reduced brain damage with a suppressive effect on microglial accumulation and interleukin-1 beta production. | Furukawa et al., 2014 |
| In addition to its previously known cognitive benefits, galantamine treatment improved quality of life in mixed dementia patients; however, the combination of galantamine and nimodipine was not advantageous. The small sample size of this study precludes any definitive conclusions. | Caramelli et al., 2014 |
| The combination of galantamine and memantine may be more effective at increasing selective cognition in schizophrenia patients than either medication alone. In the future, multitarget-directed ligands may play a role in the treatment of complex diseases like schizophrenia. | Koola et al., 2014 |
| Galantamine promotes neurogenesis by activating the M1 muscarinic and α7 nicotinic acetylcholine receptors. This study suggests that insulin-like growth factor 2 is also involved in the effects of galantamine on survival of 2-wk-old immature cells in the granule cell layer. | Kita et al., 2014 |
| Long-term treatment with galantamine significantly reduced mortality and declining cognition and daily living activities in mild to moderate AD patients. | Hager et al., 2014 |
| In addition to improving cognitive and behavioral symptoms in AD, galantamine may have disease-modifying and neuroprotective properties, as indicated by delayed amyloid β plaque formation and reduced gliosis. | Bhattacharya et al., 2014 |
| Cognition, behavior, and activities of daily living improved during 12 months of galantamine treatment. At the 3-year follow-up, a decline in all outcomes was measured; however, cognition remained higher in the treated group than that in an untreated group. | Richarz et al., 2014 |
| This study examined the galantamine-associated reversal of scopolamine-induced learning and memory impairments. | Ramakrishnan et al., 2014 |

## *Conflict of interest*

The authors declare no conflict of interest.

## Acknowledgements

This research was supported by the Bio & Medical Technology Development Program of the National Research Foundation (NRF) funded by the Ministry of Science, ICT & Future Planning (2016M3A9A5919548).

## Conflict of interest

The authors declare no conflict of interest.

## Figures and Tables

**Table 1:**
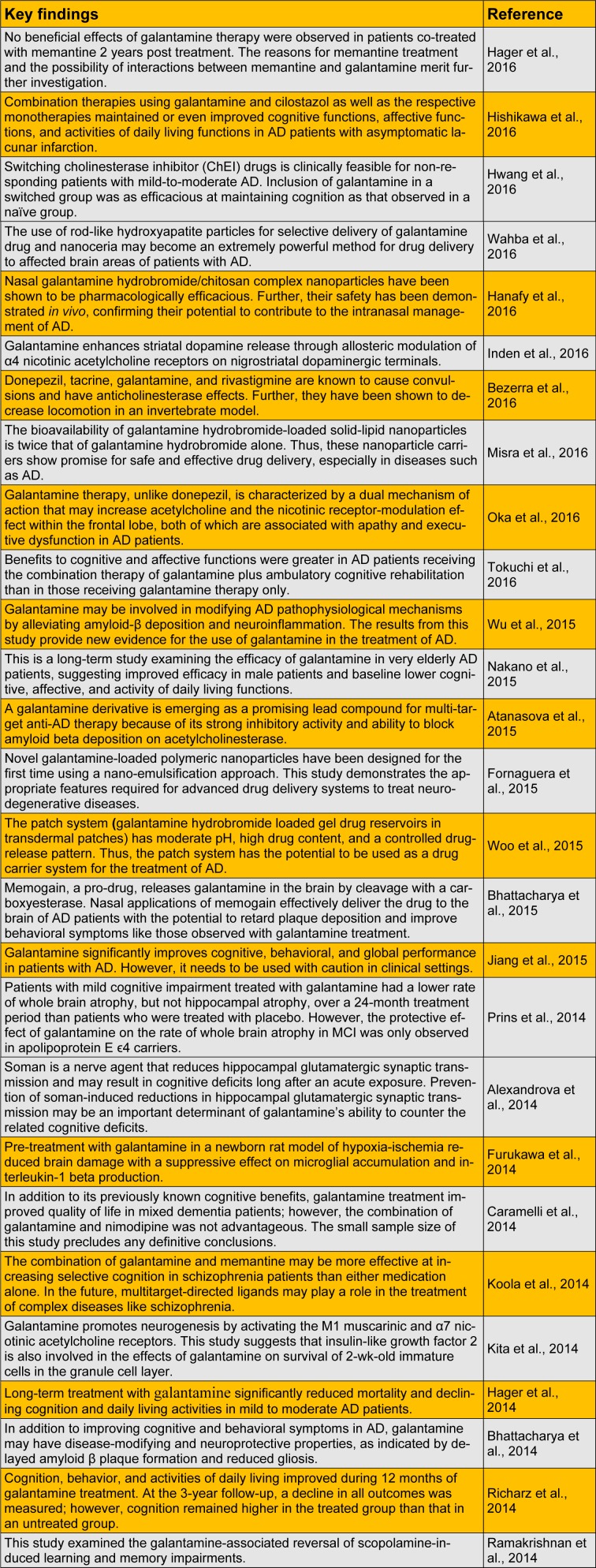
Recent studies on the pharmacological activity of galantamine
